# The Cell Surface Estrogen Receptor, G Protein- Coupled Receptor 30 (GPR30), is Markedly Down Regulated During Breast Tumorigenesis

**DOI:** 10.4137/bcbcr.s557

**Published:** 2008-04-17

**Authors:** Indira Poola, Jessy Abraham, Aiyi Liu, Josephine J. Marshalleck, Robert L. DeWitty

**Affiliations:** 1 Department of Surgery and Breast Center and; 2 Pathology, Howard University School of Medicine, Washington, DC 20059 and; 3 Biometry and Mathematical Statistics Branch, National Institute of Child Health and Human Development, National Institutes of Health, Bethesda, MD 20892

**Keywords:** breast tumorigenesis, estrogen signaling, G protein coupled receptor 30 (GPR30), cell surface estrogen receptor and lymph node metastasis

## Abstract

**Background:**

GPR30 is a cell surface estrogen receptor that has been shown to mediate a number of non-genomic rapid effects of estrogen and appear to balance the signaling of estrogen and growth factors. In addition, progestins appear to use GPR30 for their actions. Therefore, GPR30 could play a critical role in hormonal regulation of breast epithelial cell integrity. Deregulation of the events mediated by GPR30 could contribute to tumorigenesis.

**Methods:**

To understand the role of GPR30 in the deregulation of estrogen signaling processes during breast carcinogenesis, we have undertaken this study to investigate its expression at mRNA levels in tumor tissues and their matched normal tissues. We compared its expression at mRNA levels by RT quantitative real-time PCR relative to GAPDH in ERα”—positive (n = 54) and ERα”—negative (n = 45) breast cancer tissues to their matched normal tissues.

**Results:**

We report here, for the first time, that GPR30 mRNA levels were significantly down-regulated in cancer tissues in comparison with their matched normal tissues (p < 0.0001 by two sided paired t-test). The GPR30 expression levels were significantly lower in tumor tissues from patients (n = 29) who had lymph node metastasis in comparison with tumors from patients (n = 53) who were negative for lymph node metastasis (two sample t-test, p < 0.02), but no association was found with ERα, PR and other tumor characteristics.

**Conclusions:**

Down-regulation of GPR30 could contribute to breast tumorigenesis and lymph node metastasis.

## Introduction

It is now well accepted that unopposed stimulation of breast epithelial cells by the natural hormone, estrogen, plays a major role in the genesis and progression of majority of breast cancers. Although the exact molecular mechanism(s) by which estrogen promotes breast cancer progression are not known, it is presumed to be through deregulation of the complex networks of processes linked to estrogen signaling. In the normal breast epithelial cells estrogen has been linked to gene transcription through two structurally related, but distinct, high affinity receptors, ERα” and ERβ. However, ERα” seems to play insignificant role in the mature adult breast because ERα” protein is expressed only in a small fraction of normal epithelial cells. ERβ presumably plays an important role in maintaining homeostasis in breast because it is expressed in all the epithelial cells. It has become increasingly clear in recent years that a third molecule, a cell surface protein, G protein coupled receptor 30 (GPR30), mediates a number of rapid, non-genomic estrogen signaling processes and presumably contributes in maintaining the functional integrity of breast epithelial cells (1, 2).

In an effort to understand the complexity of deregulated cellular processes linked to estrogen signaling during breast carcinogenesis, we have been studying the expression of estrogen receptors in cancer tissues in comparison with normal breast tissues. We and others have shown that ERβ mRNA and protein levels were markedly diminished during breast carcinogenesis (3–5). A number of researchers have established that a majority (∼70%) of breast cancers have up-regulation of ERα” expression (5). We have undertaken the current study to understand the role of the third receptor, the cell surface estrogen receptor, GPR30, expression in breast tumorigenesis. We report here that it is markedly down regulated during breast carcinogenesis, similar to ERβ.

## Materials and Methods

HotStartTaq PCR core kits and Omniscript reverse transcriptase kits were from QIAGEN Inc. TaqMan Universal PCR Master Mix, RNAse inhibitor, random hexamers and GPR30 specific gene expression Assays-on-demand reagent (TaqMan® Gene Expression Assay ID Hs00173506_m1) were from Applied Bio-systems Inc.

### Breast tumor samples and their matched normal tissues

Breast tumor tissues and their matched normal tissues were collected from Howard University Hospital immediately after surgery and stored at −80 °C until use. Tumor samples for research were routinely harvested immediately adjacent to the histological/diagnostic section and were considered representative of the tissues used for diagnosis. Matched normal tissues were collected from a region distant from the tumor tissue of the same patient. All normal tissues used were examined by the participating pathologist and were proven to be devoid of any tumor tissue by histological staining. All the tumor samples were also examined by a pathologist and tissues containing more than 80%–90% cancer cells were excised and used for research. ERα” status in the tissues collected was determined immunohistochemically at Oncotech Laboratories using monoclonal antibodies against NH2-terminal portion of the molecule. The tumor tissues that had more than 5% positive cells were considered positive for ERα”. A total of 99 (n = 54 ERα”-positive and n = 45 ERα”-negative) cancer tissues and their matched normal tissues were included in the current study. Every normal-tumor matched pair was derived from the same patient. Tumor collection procedures were approved by the IRB at Howard University. The total number of grade I tumors were six, grade II tumors were 32, grade III tumors were 50 and the grade scoring for ten samples was not available. The total number of Stage 0 tumors were 14, Stage I tumors were 18, Stage II tumors were 40 and Stage III tumors were 12 and staging information for 15 tumors was not available. The details about the ERα” status, PR status, ages of the patients at which diagnosis were made, stage, grade, histological types, lymph node metastasis and menopausal status are given in Table 1.

### RNA extraction and cDNA synthesis

Total RNA was extracted from frozen breast tissues using Trizol reagent (Gibco-BRL Life Technologies) as previously described (3, 6, 7). RNA integrity was verified by both electrophoresis in 1.5% agarose gels and amplification of the constitutively expressed gene, glyceraldehyde-3 phosphate dehydrogenase (GAPDH). The total RNAs were reverse transcribed using Omniscript reverse transcriptase as previously described (3, 6–8).

### Conventional PCR and identification of PCR products

GPR30 was amplified by conventional PCR in an automatic thermal cycler (MJ Research, Waltham, MA) in a total volume of 12.5 μL with cDNA equivalent to 5 ng of reverse transcribed total RNA as previously described (9) using GPR30 specific sense primer, 5′ ACCAACATCTGGACGGCAGGTA 3′ (position, exon 1, 420–441 bp, NCBI accession No. NM_001505) and an antisense primer, 5′-AAGCGTGATTCTCCTTGAAG-3′ (660–679 bp, NCBI accession No. NM_001505) (10). GAPDH was amplified in parallel for only 30 cycles with cDNA reverse transcribed from 5 ng of total RNA using sense and anti-sense primers, 5′ AAGGCTGAGAACGGGAAGCTTGTCATCAAT 3′ and 5′TTCCCGTCTAGCTCAGGGATGACCTTGCCC3′, as previously described (7). The PCR amplified products (5.0 μL) were separated by electrophoresis in 1% Nu Sieve gels in Tris acetic acid EDTA buffer and detected by ethidium bromide staining. The PCR amplified products by GPR30 specific primers were purified by extracting the gel, cloned into pCR2.1-TOPO vector and the identity to GPR30 was confirmed by sequence analysis as previously described (11).

### Quantification of GPR30 mRNA by quantitative real-time PCR

Quantitative Real-Time PCRs were performed in ABI Prism GeneAmp 7900HT Sequence Detection System at a modified 50% Ramp rate as previously described (7, 12, 13). A typical real-time PCR reaction mixture contained cDNA prepared from reverse transcription of 50 ng of tumor total RNA, 1 X GPR30 specific Assays-on-Demand reagent that contains tested primers and probe and 1 X Taqman Universal PCR mix in a total volume of 20 μL. The PCR conditions were initial hold at 50 °C for two minutes, followed by denaturation for ten minutes at 95 °C, and denaturation for 15 seconds at 95 °C in the subsequent cycles and annealing and extension for 1.5 min at 60 °C for 40 cycles. All the samples were amplified in triplicate and real-time PCRs were repeated four times and normalized to the copy numbers of the housekeeping gene, GAPDH. Absolute quantification of GAPDH mRNA copy numbers was performed as previously described (12, 13). The sense, and anti-sense primers and probe for quantification of GAPDH were 5′-TTCCAGGAGCGAGATCCCT-3′, 5′GGCTGTTGTCATACTTCTTCTCATGG-3′, and FAM 5′-TGCTGGCGCTGAGTACGTCGTG-3′ TAMARA respectively.

### Statistical analysis

The mRNA expression levels of GPR-30 in tumor tissues and their matched normal tissues were compared using two-sided paired t-test. The association between the expression of GPR30 with clinicopathological parameters such as grade, stage, histological type, menopausal status and progesterone receptor status was tested by Analysis of Variance (ANOVA). The association between GPR30 mRNA levels and ERα presence was tested using two sided t-test. The association between GPR30 mRNA levels and metastasis to lymph nodes was tested using two sample t-tests. Test results were considered significant if P values were ≤0.05.

## Results

To understand the role of the newly described cell surface estrogen-binding GPR30 in tumorigenesis, we studied its expression at mRNA levels by quantitative real-time PCR. We present here the results demonstrating that GPR30 expression is significantly down-regulated in cancer tissues in comparison with their matched normal tissues. We also present here results demonstrating that GPR30 expression is significantly lower in tumors derived from patients with lymph node metastasis in comparison with tumors from patients with no lymph node metastasis. GPR30 expression levels do not correlate to any other clinico-pathological characteristics.

### GPR30 expression at mRNA levels is significantly lower in breast tumor tissues in comparison with their matched normal tissues

**Figure 1.**
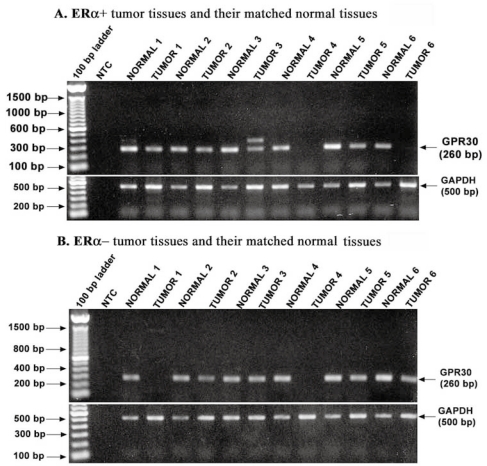
The cell surface estrogen receptor, GPR30, mRNA expression levels were decreased significantly in tumor tissues in comparison with their matched normal tissues. The tissue cDNAs were amplified for GPR30 by conventional PCR as described in Materials and Methods. Examples from six matched normal-tumor pairs in ERα-positive (A) and ERα-negative (B) tumor groups are shown. Relative levels of the house keeping gene, GAPDH, are also shown.

To understand the role of GPR30 in tumorigenesis, we first tested its expression in tumors and their matched normal tissues by conventional RT PCR. GPR30 specific primers generated 260 bp PCR product (Fig. 1). Cloning the 260 bp product into pCR2.1-TOPO vector and sequence analysis confirmed that it is the GPR30 specific sequence (data not shown). By RT and conventional PCR, we observed significantly lower GPR30 mRNA levels in tumor tissues compared to their matched normal tissues and this diminished expression varied among tumor samples. Results from six matched normal-tumor pairs from each of ERα-positive and ERα-negative tumor groups are shown in Figure 1A and Figure 1B respectively.

**Figure 2.**
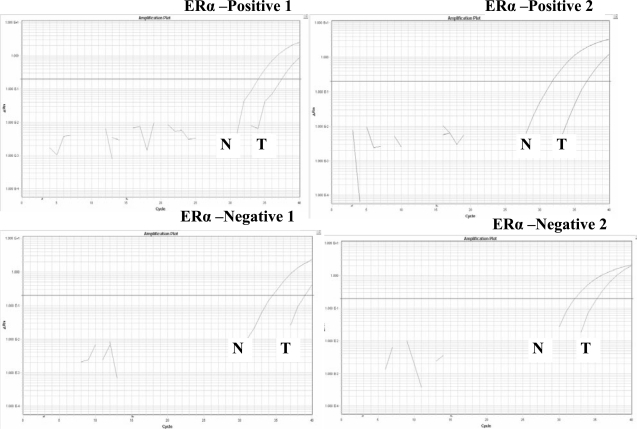
Tracings of amplification plots by RT Q real-time PCR showing expression of GPR30 in normal—tumor pairs. The RT Q real-time PCR for GPR30 was conducted as described in Materials and Methods. The mRNA expression levels were decreased significantly in tumor tissues (T) in comparison with their matched normal tissues (N). Examples of amplification plots for two matched normal-tumor pairs in ERα-positive and ERα-negative tumor groups are shown.

**Figure 3.**
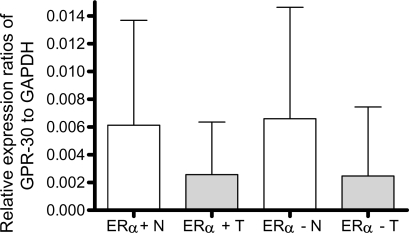
The mean expression levels of GPR30 relative to GAPDH in ERα positive and negative tumor (T) tissues and the normal (N) tissues are shown. The GPR30 levels were significantly lower in tumor tissues in comparison with their matched normal tissues (P = 0.0024 for ERα positive tissues and P < 0.0007 for ERα negative tissues, both by two-sided paired t-test). Height of the box represents the mean and height of the bar represents standard deviation above the mean.

We next quantified the GPR30 mRNA levels relative to the GAPDH levels in normal tissues and their matched tumor tissues using RT quantitative real-time PCR and comparative delta Ct method (14). By using these procedures we found that among the ERα- positive tissues, the mean GPR30 mRNA expression levels relative to GAPDH levels were 0.0058 (SD = 0.0075) in normal tissues and 0.0026 (SD = 0.0038) in cancer tissues. We next compared the relative expression levels between cancer tissues and their matched normal tissues using two- sided paired t-test. By applying this test, we found that GPR30 expression levels in cancer tissues was significantly lower than that in the normal tissues in a majority of cases (P = 0.0024; two-sided paired t-test). Among the ERα-negative samples, the relative mean GPR30 expression was 0.0067 (SD = 0.0081) in normal tissues and 0.0025 (SD = 0.0050) in cancer tissues. We observed a large standard deviation presumably because of very high variation in the individual tissues. The expression levels of GPR30 were also found to be significantly different between normal and ERα-negative cancer tissues (P < 0.0007; two-sided paired t-test). A statistically significant difference between normal and cancer tissues remains when all ERα-positive and ERα-negative tissues were combined (P < 0.0001; two-sided paired t-test). However, no significant difference was found in either normal or cancer tissue samples when we compared GPR30 mRNA levels between ERα-positive and ERα-negative tissues. (P = 0.5570 for normal samples, and P = 0.8980 for cancer samples by two-sided t-test). Quantitative data from 99 normal-tumor pairs are presented in Table 1. The mean expression values in each stage group in both ERα-positive and ERα-negative groups are shown in Table 2. Tracings of the amplification plots by Q real-time PCR for matched normal-tumor pairs two each from ERα-positive and ERα-negative groups are shown in Figure 2. The bar graphs showing the mean expression levels in tumors and normal tissues by Q real-time PCR are presented in Figure 3.

### The GPR30 mRNA levels were inversely correlated to lymph node metastasis but not to other clinico-pathological characteristics

**Figure 4.**
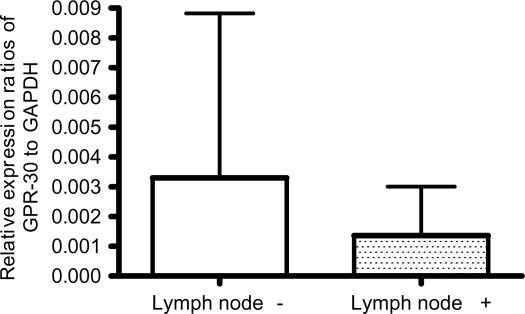
The mean expression levels of GPR30 relative to GAPDH in tumor tissues derived from patients who were positive (+) and negative (−) for lymph node metastasis are shown. The GPR30 levels were significantly lower in lymph node positive tumors compared to negative tumors (P < 0.02 by two sample t-test). Height of the box represents the mean and height of the bar represents standard deviation above the mean.

After establishing that GPR30 levels were significantly lower in cancer tissues compared to their matched normal tissues, we next tested whether any correlation exists between its levels and clinico-pathological characteristics by analysis of variance (ANOVA). When we compared the GPR30 values between three tumor grades the mean values obtained for Grade I, Grade II and Grade III were 0.0040, 0.0029 and =0.0023 respectively. By ANOVA we did not find any statistically significant difference in GPR30 levels among the cancer tissues of different grades (P-values for testing null hypothesis (H0): g1 = g2, g2 = g3, g3 = g1 were 0.6046, 0.5966, and 0.4182 respectively). Similarly, the mean expression values for Stage 0, I, II and III were 0.0033, 0.0047, 0.0018 and 0.0013, respectively, and no statistically significant differences were found among samples of different stages by ANOVA (P-values for testing H0: t1 = t2, t1 = t3, t1 = t4, t2 = t3, t2 = t4, t3 = t4 were 0.3677, 0.3029, 0.2717, 0.0258, 0.0452, and 0.7314 respectively). We also did not find any association between GPR30 levels with menopausal status, progesterone status, ages of the patients at diagnosis, ERα, or histological type of the tumor. However, we found a significant association with tumor metastasis to lymph nodes. The cancer tissues from patients who were negative for lymph node metastasis (n = 53) had significantly higher expression (mean = 0.0033, SD = 0.0055) than tumors from patients who were positive for lymph node metastasis (n = 29) (mean = 0.0014, SD = 0.0016) (Table 3). Again, we observed a large standard deviation presumably due to very high variation in individual samples. The P-value by a two-sample t-test was <0.02. Bar graphs showing the mean expression levels in tumors from patients who were positive and negative for lymph node metastasis are presented in Figure 4.

## Discussion

For several years there has been a speculation that estrogen could be binding to a cell surface membrane protein independent of ERα and ERβ (15, 16). In recent years several independent research teams have identified GPR30 as the membrane protein that binds estrogen (17–19). GPR30 belongs to the super family of over 1000 of seven-transmembrane spanning cell surface orphan receptors that are known to bind agonists such as chemokines, vasoreactive substances and neurotransmitters (20).

It is now fairly well recognized that GPR30 binds estrogen and the complex is internalized to trigger a variety of rapid non-genomic estrogen signaling events that include increasing the second messenger levels such as Ca^+^ mobilization (21), NO generation (22), intracellular phosphatidyl inositol 3, 4, 5 triphosphates (23), cAMP (24) and activation of MAP kinases, Erk1 and Erk2 (25). Data are emerging on the details about the non-genomic actions of estrogen on cellular events and cross-talk with growth factor mediated Erk1 and Erk2 activation through release of cell surface associated EGF and activation of EGFR tyrosine kinase activity (26). It appears that through GPR30, estrogen triggers opposing G protein dependent signaling mechanisms that act to balance MAPK signaling cascade events, Erk1 and Erk2 activation (27). Since a number of growth signaling molecules use MAPK cascade and cross-talk with ERα to increase cell proliferation, the dual regulation of Erk1/2 activation by GPR30 suggests that a coordinated signaling between GPR30 and ERα is required for controlled growth and regulation of normal epithelial cells. In addition to estrogen, the proliferation opposing progestins also seem to use GPR30 for their actions (28). It has been reported that continuous administration of progestins inhibit breast cancer cell proliferation and GPR30 is critical for this growth inhibition (29). All these reports suggest that GPR30 could play a critical role in hormonal regulation of breast epithelial cell integrity. Deregulation of the events mediated by GPR30 could contribute to tumorigenesis and influence clinicopathological behavior of breast cancer.

As a first step in understanding the role of GPR30 in the deregulation of estrogen signaling processes during breast carcinogenesis, we have undertaken this study to investigate its expression at mRNA levels in tumor tissues and their matched normal tissues. Previously, GPR30 expression was investigated by Weigel’s group (10) at mRNA levels in seven ERα-negative and four ERα-positive breast cancer tissue by conventional RT PCR. They reported the presence of GPR30 mRNA in one ERα-positive and all four ERα-negative breast cancer tissues. However, there were no data on its expression in normal breast tissues and the number of tissues analyzed was too small to draw statistically significant conclusions. Ravanakar et al. (17) reported GPR30 expression in breast cancer cell lines. Filardo et al. studied the expression of GPR30 protein levels in 12 reduction mammoplasty and 361 breast cancer tissues by immunohistochemistry. They reported its presence in all reduction mammoplasty and 62% of cancer tissues and an association with ERα, Her2/neu and tumor size and inverse correlation with metastasis. However, their study did not establish its role in breast tumorigenesis because their control (normal) tissue samples were very few, not matched with the tumor samples, and the assay employed was not quantitative (30).

In the current study, to understand the role of GPR30 in tumorigenesis, we studied its expression in matched normal-tumor tissue pairs, each pair derived from the same patient. We employed a quantitative approach to determine the levels of GPR30 mRNA by RT quantitative real-time PCR with reference to the levels of a house keeping gene, GAPDH. The data presented here on 99 matched normal-tumor pairs demonstrate that GPR30 gene expression is markedly down regulated in cancer tissues in comparison with their matched normal tissues. We observed diminished expression of GPR30 mRNA in most of the cancer tissues in comparison with their respective matched normal tissues (n = 99, p < 0.0001 by two sided paired t-test). Our results suggest that tumorigenesis may arise, in part, by the loss of GPR30. However, Maggiolini et al. have shown that GPR30 activation in breast, ovarian and endometrial cancer cell lines triggers signaling pathways such as MAPK pathway involved in cell proliferation (31, 32, 33). Perhaps, the opposing effects of GPR30 on the EGF-receptor mediated MAPK pathway may explain its role both in transformation and inhibition as demonstrated by Filardo et al. (34).

Although the decrease was observed in a majority of tissue pairs, it varied from one matched pair to the other (Table 1 and Table 2) and the variation did not correlate to tumor size, grade, stage, age at diagnosis, menopausal status, and histological type. The down-regulation of GPR30 during breast carcinogenesis also appears to be independent of ERα protein expression (P = 0.898, by two sided t-test, n = 54 ERα”—positive and n = 45 ERα”—negative). The decreased levels of GPR30 during breast carcinogenesis, independent of ERα and other clinicopathological characteristics of tumors, are similar to ERβ expression. We and several other researchers have reported lack of association of ERβ levels with tumor characteristics and ERα expression (7,35–36).

Although the variation of decreased GPR30 expression did not correlate to tumor size, ERα, grade, stage, menopausal state, age and histological type, we observed a much marked decreased expression in tumor tissues from patients who were positive for lymph node metastasis compared to the tumor tissues from patients who were negative for lymph node metastasis (Table 1, Table 3 and Fig. 4) (Two sample t-test, P < 0.02, n = 29 node positive and n = 53 node negative). Our results on inverse correlation of GPR30 expression with lymph node metastasis are in agreement with the report by Filardo et al. (30). Because of the inverse association with lymph node metastasis, the relative levels of GPR30 to GAPDH could be applied as a marker to determine the cancer cell invasion.

## Figures and Tables

**Table 1.** Relative ratios of GPR30 transcripts to GAPDH in breast cancer tissues and their matched normal tissues.

**#**	**Age**	**Menopausal state**	**Stage**	**Grade**	**Nodal status**	**# of nodes affected**	**Histological type**	**ER status**	**PR status**	**Ratio of GPR-30 to GAPDH in normals**	**Ratio of GPR-30 to GAPDH in tumors**
1	35	Pre	2B	III	+	1/7	Ductal carcinoma	+	−	0.003725	0.000249
2	84	Post	2A	II	−	0	Ductal and lobular carcinoma	+	+	0.001567	0.000225
3	77	Post	2A	II	−	0/16	Ductal carcinoma	+	−	0.006243	0.000613
4	77	Post	3A	III	+	24/24	Ductal carcinoma	+	−	0.003339	0.001205
5	82	Post	2A	NA	−	0/7	Invasive Papillary carcinoma	+	+	0.001096	0.000638
6	48	Pre	2A	III	−	0/13	Ductal carcinoma	+	+	0.001225	0.001063
7	37	Pre	2A	II	−	0/17	Ductal carcinoma	+	NA	0.002443	0.001406
8	64	Post	0	NA	−	0	Papillary carcinoma	+	NA	0.001944	0.001287
9	60	Post	I	NA	−	0/97	Adenocarcinoma	+	−	0.00139	0.00014
10	65	Post	I	II	−	0/97	Ductal carcinoma	+	−	0.001425	0.002243
11	66	Post	0	III	−	0/3	DCIS	+	NA	0.00162	0.001618
12	50	Post	2B	III	+	1	Ductal carcinoma	+	−	0.004109	0.001142
13	59	Post	0	I	−	0	Ductal and lobular carcinoma	+	+	0.007525	0.01277
14	77	Post	3B	I	+	02/05	Ductal carcinoma	+	+	0.01656	0.006584
15	55	Post	0	II	−	0/7	DCIS	+	−	0.031082	0.003393
16	45	Pre	I	II	−	0/5	Ductal carcinoma	+	−	0.020564	0.021204
17	53	Post	0	III	−	0	DCIS	+	NA	0.008429	0.008225
18	45	Pre	0	I	−	0/13	DCIS	+	+	0.001114	0.001321
19	73	Post	0	II	−	0	DCIS	+	NA	0.00968	0.002364
20	67	Post	3A	II	+	6/18	Ductal carcinoma	+	−	0.003209	4.8E-05
21	86	Post	2	III	+	3/7	Ductal carcinoma	+	+	0.005889	0.000282
22	66	Post	2A	I	−	0/11	Ductal carcinoma	+	+	0.03154	0.002227
23	32	Pre	2A	II	NA	NA	Ductal carcinoma	+	+	0.014706	0.00243
24	40	Pre	0	III	−	0/15	DCIS	+	NA	0.006445	0.002679
25	39	Pre	2B	II	+	12/12	Ductal carcinoma	+	−	0.000855	0.000117
26	74	Post	2B	III	+	1/18	Ductal carcinoma	+	−	0.001437	0.001636
27	60	Post	2A	III	−	0/18	Mucinous carcinoma	+	−	0.002934	0.005897
28	50	Pre	2A	NA	−	0/13	Lobular carcinoma	+	+	0.001379	0.000941
29	72	Post	I	III	+	5/26	Ductal carcinoma	+	−	0.000847	0.00127
30	46	Pre	0	III	−	0	DCIS	+	−	0.006932	0.007201
31	90	Post	II	III	−	0/6	Ductal carcinoma	+	−	0.000764	0.000779
32	39	Pre	0	II	−	0	DCIS	+	NA	0.003122	0.001061
33	65	Post	II	NA	+	8/13	Lobular carcinoma	+	−	0.002103	0.004043
34	49	Pre	II	III	−	0/14	Ductal carcinoma	+	+	0.010478	0.00222
35	83	Post	NA	III	−	0/3	Ductal carcinoma	+	+	0.003061	0.00142
36	42	Pre	0	1	−	0	DCIS	+	NA	0.003694	0.000552
37	70	Post	2B	II	+	2/16	Ductal carcinoma	+	−	0.000644	0.004956
38	43	Pre	IIIA	II	+	7/8	Ductal carcinoma	+	+	0.003416	0.001362
39	58	Post	0	NA	−	0/16	DCIS	+	NA	0.000918	0.000251
40	42	Pre	IIIA	I	NA	NA	Ductal carcinoma	+	+	0.002145	0.000388
41	88	Post	NA	II	+	3/10	Ductal carcinoma	+	+	0.003182	0.000128
42	63	Post	NA	II	NA	NA	Ductal carcinoma	+	−	3.07E-05	0.012541
43	68	Post	IIA	II	−	0/18	Ductal carcinoma	+	+	0.000502	0.002865
44	50	Post	NA	II	NA	NA	Ductal carcinoma	+	+	0.016977	0.000215
45	61	Post	0	NA	−	0	DCIS	+	NA	0.004196	0.000343
46	40	Pre	IIA	II	+	7/8	Ductal carcinoma	+	+	0.000998	0.000128
47	57	Post	2A	III	+	1/9	Ductal carcinoma	+	−	0.028543	0.000557
48	47	Pre	2B	III	+	5/16	Ductal carcinoma	+	−	0.00227	0.003588
49	90	Post	3B	II	+	0	Ductal carcinoma	+	−	0.005614	0.001245
50	73	Post	I	II	−	0 /13	Ductal carcinoma	+	NA	0.004111	0.002814
51	91	Post	2A	II	−	0	Ductal carcinoma	+	−	0.002508	0.001272
52	42	Pre	NA	III	NA	NA	Ductal carcinoma	+	+	0.005298	0.000904
53	72	Post	NA	NA	NA	NA	Carcinoma	+	NA	0.001606	0.00131
54	67	Post	2B	II	−	0/15	Ductal carcinoma	+	−	0.006579	0.001448
55	52	Pre	I	III	−	0/27	Ductal carcinoma	−	+	0.020204	0.000215
56	62	Post	3B	III	+	7/23	Ductal carcinoma	−	−	0.0031	0.000175
57	46	Pre	NA	III	NA	NA	Ductal carcinoma	−	+	0.00391	0.003327
58	70	Post	2A	II	−	0/97	Ductal carcinoma	−	−	0.020928	0.003574
59	42	Pre	2B	III	−	0/9	Ductal carcinoma	−	−	0.001886	0.00118
60	62	Post	2A	NA	−	0/17	Medullary Carcinoma	−	−	0.006263	0.00188
61	52	Post	I	II	−	0/50	Ductal Carcinoma	−	−	0.001624	0.005946
62	55	Post	3A	III	+	4/21	Ductal Carcinoma	−	−	0.00043	0.001517
63	59	Post	2B	III	−	0	Ductal Carcinoma	−	−	0.024845	0.004184
64	53	Post	2A	II	−	0/10	Ductal Carcinoma	−	−	0.002734	0.005019
65	47	Pre	I	NA	−	0/18	Comedo Carcinoma	−	−	0.003812	0.001801
66	38	Pre	0	III	−	0	DCIS	−	−	0.005063	0.00256
67	57	Post	3A	III	+	17/17	Ductal carcinoma	−	−	0.00539	0.001154
68	49	Pre	2A	III	−	0	DCIS	−	−	0.006231	0.000757
69	85	Post	I	III	−	0	Ductal carcinoma	−	−	0.001793	0.000467
70	56	Post	I	III	−	00/22	Ductal carcinoma	−	−	0.005093	0.000733
71	40	Post	2B	III	+	+	Ductal carcinoma	−	−	0.000805	0.000185
72	64	Post	2A	III	−	0	Ductal carcinoma	−	−	0.003861	0.000852
73	67	Post	2A	III	NA	NA	Ductal carcinoma	−	−	0.00197	0.000134
74	34	Pre	2B	III	NA	NA	Ductal carcinoma	−	−	0.014494	0.000957
75	56	Post	2B	III	−	0/5	Ductal carcinoma	−	−	0.032787	0.005191
76	85	Post	2B	II	+	11/15	Ductal carcinoma	−	−	0.003897	0.001344
77	70	Post	I	II	−	0/34	Carcinoma	−	−	0.000853	0.000215
78	27	Pre	3A	III	+	4/6	Ductal carcinoma	−	−	0.004194	0.000252
79	70	Post	I	II	−	0/23	Ductal carcinoma	−	−	0.004126	0.004248
80	70	Post	I	III	−	0/11	Ductal carcinoma	−	−	0.009754	0.00184
81	73	Post	1	II	−	0	Ductal carcinoma	−	−	0.003225	0.002522
82	39	Pre	II	III	+	1/24	ductal carcinoma	−	−	0.001684	0.000118
83	51	Pre	III	III	+	7/10	ductal carcinoma	−	−	0.001289	0.000222
84	51	Post	I	II	−	0/2	Ductal carcinoma	−	+	0.004348	0.00494
85	77	Post	II	III	+	1/15	Ductal carcinoma	−	−	0.000335	0.001718
86	57	Post	NA	III	+	1/11	Duct carcinoma	−	−	0.005029	0.000203
87	80	Post	NA	III	NA	NA	Ductal carcinoma	−	−	0.003582	0.002373
88	60	Post	3B	III	NA	NA	Ductal carcinoma	−	−	0.000449	0.001562
89	60	Post	I	III	−	0	Ductal carcinoma	−	+	0.019322	0.033725
90	40	Pre	II	III	NA	NA	Ductal carcinoma	−	−	0.000297	0.001392
91	45	Pre	NA	III	NA	NA	ductal carcinoma	−	−	0.013692	0.003294
92	56	Post	NA	III	NA	NA	Ductal carcinoma	−	−	0.001469	0.000942
93	47	Pre	NA	II	+	13/13	Ductal carcinoma	−	−	0.000416	0.00061
94	33	Pre	NA	III	NA	NA	Ductal carcinoma	−	−	0.010816	0.000427
95	47	Pre	I	III	−	0	Ductal carcinoma	−	−	0.002625	1.04E-05
96	60	Post	IA	II	−	0/2	Ductal carcinoma	−	−	0.00161	0.000347
97	44	Pre	NA	III	NA	NA	Ductal carcinoma	−	−	0.000105	9.78E-05
98	54	Post	2	III	+	1/12	Ductal carcinoma	−	−	0.028366	0.003486
99	53	Pre	NA	NA	NA	NA	Carcinoma	−	−	0.014414	0.002844

**Abbreviation:** NA: Not Available

**Table 2.** Summary of the specimens used and the mean GPR30 expression values.

**ERα Status of tumors**	**# of specimens studied**	**Mean (SD) of GPR30 Expression level (relative to GAPDH) in cancer tissues**	**Mean (SD) of GPR30 Expression level (relative to GAPDH) in normal tissues**
ERα-positive	54	0.0026 (0.0038)	0.0058 (0.0075)
Stage 0	13	0.0033 (0.0038)	0.0067 (0.0079)
Stage I	5	0.0055 (0.0088)	0.0057 (0.0084)
Stage II	24	0.0017 (0.0016)	0.0056 (0.0083)
Stage III	6	0.0018 (0.0024)	0.0057 (0.0054)
Stage IV	0	0	0
Stage	6	0.0028 (0.0048)	0.0050 (0.0061)
Not available			
ERα-negative	45	0.0025 (0.0050)	0.0067 (0.0081)
Stage 0	1	0.0026 (NA)	0.0051 (NA)
Stage I	13	0.0044 (0.0090)	0.0060 (0.0065)
Stage II	16	0.0020 (0.0017)	0.0095 (0.0111)
Stage III	6	0.0008 (0.0007)	0.0025 (0.0021)
Stage IV	0	0	0
Stage	9	0.0016 (0.0014)	0.0059 (0.0056)
Not available			

**Table 3.** The relative GPR-30 expression levels to GAPDH (mean and standard deviation) in tumor tissues derived from patients with or without tumor metastasis to lymph nodes.

	**Positive Lymph Node Metastasis**	**Negative Lymph Node Metastasis**
Mean	1.363 × 10^−3^	3.296 × 10^−3^
Standard Deviation	1.641 × 10^−3^	5.529 × 10^−3^

## Abbreviations

GAPDH: Glyceraldehyde-3 phosphate dehydrogenase;
GPR30: G-protein coupled receptor 30;
ERα”,: estrogen receptor alpha;
ERβ: Estrogen receptor beta; and
PR: Progesterone receptor.
